# Dual Effects of Cellular Immunotherapy in Inhibition of Virus Replication and Prolongation of Survival in HCV-Positive Hepatocellular Carcinoma Patients

**DOI:** 10.1155/2016/6837241

**Published:** 2016-03-16

**Authors:** Lei Qian, Nanya Wang, Huimin Tian, Haofan Jin, Hengjun Zhao, Chao Niu, Hua He, Tingwen Ge, Wei Han, Jifan Hu, Dan Li, Fujun Han, Jianting Xu, Xiao Ding, Jingtao Chen, Wei Li, Jiuwei Cui

**Affiliations:** ^1^Department of Cancer Center, The First Hospital of Jilin University, Changchun 130021, China; ^2^Institute of Translational Medicine, The First Hospital of Jilin University, Changchun 130021, China

## Abstract

Immune cells play an important role in the development and progression of hepatitis C virus (HCV) and hepatocellular carcinoma (HCC). We conducted a retrospective study to evaluate the influence of adoptive cellular immunotherapy (CIT) on viral load and progression-free survival (PFS) for HCC patients infected with HCV. Patients (*n* = 104) were divided into a control group (conventional therapy, *n* = 73) and study group (combination of CIT and conventional therapy, *n* = 31). Autologous mononuclear cells were induced into natural killer, *γδ*T, and cytokine-induced killer cells and infused intravenously to study group patients. More patients had shown viral load decrease or were stable in study group (100% versus 75%) (*p* = 0.014). The median PFS of the study group and control group was 16 and 10 months, respectively (*p* = 0.0041), and only CIT was an independent prognostic factor for PFS (hazard ratio, 0.422; *p* = 0.005). Three patients developed transient moderate fever after infusion, and there were no significant differences in alanine aminotransferase and aspartate aminotransferase levels before and after treatment in both groups. Our results show that CIT contributes to improvement of prognosis and inhibition of viral replication in HCV-related HCC patients, without impairment of liver function.

## 1. Introduction

Hepatocellular carcinoma (HCC) is the sixth most common cancer worldwide, with 749 000 new cases diagnosed in 2008, and the third-ranking cause of cancer-related deaths, contributing to 695 000 cases annually. HCC is mainly caused by hepatitis B virus (HBV) and hepatitis C virus (HVC) infection. It has been estimated that over 185 million people are chronically affected with HCV worldwide. Furthermore, chronic hepatitis C patients show a higher probability of developing liver cirrhosis and HCC, which represents a significant public health problem [1, 2].

Accordingly, virus elimination or control is important for reducing the rate of tumor occurrence in hepatitis C patients [3, 4], which may also contribute to protecting liver function and decreasing the risk of recurrence in the context of HCC. Currently, the combination of pegylated interferon-*α* and ribavirin is the recommended treatment for chronic hepatitis C; however, this therapy has frequent and numerous side effects, especially for those in the decompensation stage of liver cirrhosis and HCC [5]. The introduction of novel nucleotide analogues such as sofosbuvir has brought about a new treatment era for HCV patients; however, these drugs remain very expensive and do not appear to be suitable for hepatitis C patients with HCC [6, 7]. Thus, at present, there is essentially no effective drug available for HCC patients with HCV for controlling virus replication. Furthermore, few studies focused on the relationship between the HCV load and outcome of HCC patients partly due to limited option of drugs suitable for hepatitis C virus control in HCC, although it is well confirmed that control of HBV contributes to decreasing recurrence of HCC.

The intrahepatic immune system is likely to play a key role in determining the outcome of HCV infection, because of its potential for viral clearance [8]. The hepatic lymphocyte repertoire is characterized by high CD8/CD4 T cell ratios and large numbers of gamma delta T (*γδ*T) cells and natural killer (NK) cells. Persistence of HCV is generally considered to be due to qualitative and/or quantitative inadequacies in these cells, which influences the immune response [9]. Many studies have also demonstrated impaired T cell activity in HCV-infected patients, and viral persistence has been attributed to defective T cell immunity [8]. Recently, the role of innate immunity in determining the outcome of HCV infection and in regulating and maintaining specific immune responses has received increasing attention [10, 11]. For example, Corado et al. [12] showed that spontaneous NK cytotoxicity was fourfold lower in HCV patients than in controls and suggested that altered NK cell function may be a significant contributing factor to the chronicity of HCV infection. In fact, the clearance or control of HCV depends on the synergistic effect of various immunocytes.

Most of the previous work on immunity against HCV has focused on the generation of hepatic HCV-specific T cell lines* in vitro* [13]; however, recent studies have highlighted essential roles for NK, *γδ*T, and cytokine-induced killer (CIK) cells. These cells not only show direct antitumor or antivirus effects but are also required for the optimal priming and cytotoxic function of specific T cells [14–16]. In addition to the similar antitumor and antivirus effects of these three kinds of innate immune cells, they also have synergistic effects and are influenced differently by the intrahepatic environment and HCV virus. For example, intrahepatic *γδ*T and T cell activation could be directly induced by the HCV/E2 particle through CD81 triggering. By contrast, NK cells might be inhibited by the HCV/E2 particle [17]. Therefore, this combination could be useful to overcome immune resistance in various aspects.

Indeed, in a preliminary study, we observed that the hepatitis C viral load declined in three patients diagnosed with HCC that received a combination of cellular immunotherapy (CIT) with NK, *γδ*T, and CIK cells along with conventional radiofrequency ablation (RFA) for HCC [18]. Although liver dysfunction was not observed in our previous study and autologous CIT have been reported to be well-tolerant, the potential side effect merits consideration. Therefore, in this retrospective study, we investigated whether the combination of NK, *γδ*T, and CIK cells might inhibit HCV replication in HCC patients and the specific effects of this treatment on progression-free survival (PFS). In addition, we carefully monitored the change in liver function and other events after infusion.

## 2. Materials and Methods

### 2.1. Patients and Study Design

**Figure 1:**
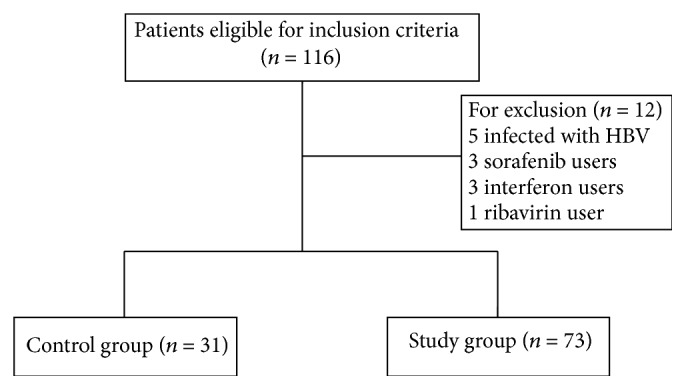
Flowchart for inclusion and exclusion details.

The HCC patients infected with HCV that were hospitalized from January 2010 to March 2015 in the Cancer Center of the First Hospital of Jilin University were retrospectively analyzed in the study. The inclusion criteria were as follows: (1) diagnosis of HCC by biopsy/imaging; (2) presence of HCV infection confirmed by Real-Time PCR Hepatitis C Virus RNA Diagnostic Kit; (3) patients with newly diagnosed or recurrent HCC; (4) Eastern Cooperative Oncology Group (ECOG) performance status ≥ 3; (5) obtaining complete remission (CR), partial remission (PR), or stable disease (SD) after conventional therapy (surgery, RFA, transcatheter arterial chemoembolization [TACE]); (6) giving CIT after conventional therapy and within three months, in case of patients receiving CIT. The exclusion criteria were as follows: (1) interferon and/or ribavirin use during the study period; (2) concomitant infection with human immunodeficiency virus or HBV; (3) use of sorafenib (see Figure 1). Patients were divided into the study group (CIT combined with conventional therapy) and control group (conventional therapy alone).

The baseline characteristics, change of viral load, and alanine aminotransferase (ALT) and aspartate aminotransferase (AST) levels were collected and compared between groups. We defined AST1/ALT1 as the first point of detection before treatment and AST2/ALT2 as the second point of detection after treatment. Thus, the change of liver function was measured according to deviation in these values (denoted as “AST2-ASL1” or “ALT2-ALT1”). PFS was defined from the time of conventional therapy (RFA, TACE, or surgery) to progression. The data of viral load and ALT/AST used for comparison were selected from the measurements taken close to these two points of time used for PFS determination. The primary and secondary endpoints of this study were viral load change and PFS, respectively.

The combination of conventional treatment and CIT for HCC patients was an observational study in our hospital, and the study design was previously reviewed and approved by the Ethics Committee of the First Hospital of Jilin University, and informed consent was obtained from each patient.

### 2.2. Immunocytes Preparation and Infusion

Autologous peripheral blood mononuclear cells (PBMCs) (1–1.5 × 10^9^ cells) were obtained from HCC patients by apheresis using COBE SPECTRA*™* (Gambro BCT, Inc., Lakewood, CO) (D0). After collection, PBMCs were split into two 50 mL centrifuge tubes that were spun for 10 min at 3000 rpm. The supernatant was discarded, and the cell pellets were resuspended in 30 mL of phosphate buffered saline (PBS) and placed on top of a 15 mL Hypaque (Amersham Biosciences) in a 50 mL sterile tube. Lymphocytes were isolated from PBMCs by means of Ficoll-Hypaque density centrifugation (Ficoll separation) to yield ~1.5 × 10^9^ (1.1–1.8 × 10^9^) cells and were then separated into three pools to induce NK, *γδ*T, and CIK cells through the use of different cytokines as previously described [18]. All procedures for preparing the autologous immune cells were carried out under good manufacturing practice conditions, approved by the Jilin Provincial Center for Sanitation Inspection and Test (China, certificate ID A20090047).

Before administration, immunocytes stained with specific fluorescence-conjugated monoclonal antibodies (mAbs) (BD Biosciences, San Diego, CA, USA) were identified via four-color flow cytometry performed on a FACSCalibur system (BD Biosciences, San Diego, CA, USA) [18]. NK cells were then incubated for 15 min with CD3-PerCP, CD69-PE, and CD56-APC. CIK cells were then incubated with CD3-PerCP, CD4-FITC, CD8-PE, and CD56-APC. Finally, *γδ*T cells were incubated with V*γ*9-FITC and CD3-APC.

The release criteria for the cultured cells were as follows: (i) cell viability > 90%; (ii) no contamination of mycoplasma, endotoxin, bacteria, or fungi, as determined by PCR, performed 24 h before and on the day of product release; (iii) the number of the cells around 1-2 × 10^9^ per infusion; (iv) the percentage of NK and *γδ*T cells each >50%, as detected by flow cytometry.

The initial transfusion began 14 days after apheresis. One course of CIT was accomplished during D14–D17 after the apheresis, including infusion of NK, CIK, and *γδ*T cells. Each patient received 8 infusions (2 infusions per day) in one course of CIT.

### 2.3. HCV RNA Determination

The HCV viral load in serum was detected by the Real-Time PCR Hepatitis C Virus RNA Diagnostic Kit (Shanghai Haoyuan Biotech Co, Ltd.). The change in viral load was measured by subtracting the before-treatment value from the after-treatment value, with the HCV RNA values transformed to the log scale. For further statistical comparison, patients were classified into different categories according to the degree of change in HCV RNA as follows: increase over 1 log, increase between 0.5 and 1 log, increase between 0 and 0.5 log, decrease over 1 log, decrease between 0.5 and 1 log, and decrease between 0 and 0.5 log. If the change was between 0 and 0.5 log in either direction, the viral load was regarded as stable. Otherwise, the change was regarded as a meaningful change, that is, representing an actual decrease or increase.

### 2.4. Adverse Events

We evaluated the adverse events by monitoring and talking to patients during the infusion. In general, previous reports have shown that transfusion of immune cells may cause fever, rash, or arthralgia. Thus, the evaluation included all of the aforementioned symptoms in addition to any other main complaints from the patients. Given that immunocytes may suppress HCV replication through killing infected hepatocytes, which could lead to the release of hepatic transaminase, ALT and AST levels were detected before and after CIT. The adverse events were evaluated according to Common Terminology Criteria for Adverse Events (CTCAE) version 3.0.

### 2.5. Statistical Analysis

The chi-square test was used to compare baseline clinical characteristics, and the nonparametric Mann-Whitney test was used for comparing the difference in HCV RNA and ALT/AST levels in the two groups. The Spearman test was used to explore the correlation between a change in viral load and PFS. PFS analysis was conducted using the Kaplan-Meier and Cox proportional hazards models (SPSS 17.0, Chicago, IL). A two-tailed *p* value less than 0.05 was considered statistically significant.

## 3. Results

### 3.1. Clinical Characteristics of the Patients

The general clinical characteristics of the patients are summarized in Table 1. A total of 104 eligible patients were included in the study: 73 in the control group and 31 in study group. The records of the viral loads for 8 patients were incomplete (3 in the control group, 5 in the study group); thus, these data were regarded as missing values in the statistical analysis.

The median age was 65 years (range, 46–82). The majority of the patients were male (61/104, 59%), with stage A HCC (56/104, 54%) according to Barcelona Clinic Liver Cancer (BCLC) staging system. The conventional therapies were mainly determined according to the Chinese Guideline on HCC published in 2009. For all patients, routine clinical examinations and evaluations were completed within 4 days after hospitalization. The strategy of treatment was determined by multiple disciplinary team (MDT) in our hospital. Usually, RFA therapy was used for patients who had single or two tumors less than 3 cm diameter without blood vessel invasion and metastasis. Patients who had tumors less than 4 and tumor diameter less than 5 cm with or without vessel invasion (most with classification A and partly with BCLC stage B) received surgery. TACE treatment was chosen for patients who had more progressive stage with the Child-Pugh score A or B. Doxorubicin Hydrochloride (20–50 mg) and Lipiodol (5–10 mL) were used for TACE treatment.

The baseline characteristics, including age, gender, BCLC stage, and initial therapy, were well-balanced between the study and control groups, with no significant differences according to the chi-square test, indicating the suitability of these groups for further analysis (Table 1). In addition, for the study and control group, the median AST level was 49 IU/mL (range, 21–102 IU/mL) and 56 IU/mL (range, 20–313 IU/mL), indicating no significant difference (*p* = 0.215) (Table 2). For the study group, the median number of CIT courses was 3 (range, 1–11).

### 3.2. Quality of Cultured Immunocytes

**Figure 2:**
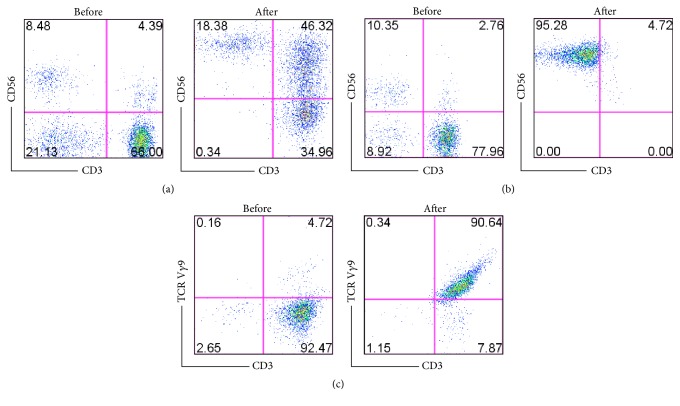
(a) The percentage of CIK cells including (CD3+ CD56+), (CD3+ CD56−), and (CD3− CD56+) before and after induction and CD4+ and CD8+ before and after induction in one of the patients. (b) The percentage of NK cells (CD3+ CD56−) before and after induction and the activated NK (CD56+ CD69+) before and after induction in one of the patients. (c) The percentage of *γδ*T before and after induction in one of the patients.

The immunocytes were induced and expanded successfully in all patients. The viability of the immunocytes was found to exceed 95%. Mycoplasma, endotoxin, bacteria, and fungi were detected to confirm no contamination. The percentages of CIK (CD56+CD3+), NK (CD56+CD3+), and *γδ*T (V*γ*9+) cells before and after induction were 4.39% (1.5–8%) versus 46.32% (27–50%), 10.35% (5.1–12.6%) versus 95.28% (70.1–99.6%), and 4.72% (2.61–11.2%) versus 90.64% (60.5–97.9%), respectively. Representative results from one patient in the study group are shown in Figure 2. The cytotoxicity of expanded immunocytes to cancer cell line (HepG2) was detected by LDH release assay* in vitro* as described in our previous study [18].

### 3.3. Decline in Viral Load

In the study group, 4 of 28 patients showed a decrease in viral load greater than 1 log, whereas no patient had an increase greater than 1 log. By contrast, in the control group, 5 of 68 patients showed a decrease in HCV load greater than 1 log, while 9 patients showed an increase greater than 1 log (Table 3). Thus, considering a change of ±0.5 log as stable, overall, 17/68 (25%) of the patients showed an increase in viral load, 37/68 (54%) were stable, and 14/68 (21%) showed a decrease in the control group. In contrast, no patient in the study group showed an increase in viral load, 20/28 (71%) were stable, and 8/28 (29%) showed a decrease (Table 4). There was a significant difference between the two groups according to the nonparametric Mann-Whitney test (*p* = 0.014).

Furthermore, we considered the possibility that there might be a difference in the reaction to immunotherapy between sexes, given that gender has been shown to be significant predictive factor of the efficacy of interferon treatment in chronic hepatitis C patients. Therefore, we also compared the change in viral load between male and female patients in the study group. Among the patients who showed a viral load decrease in the study group, 5/13 (38.4%) were female and only 3/18 (16.7%) were male (*p* = 0.028).

### 3.4. Prolongation of PFS

**Figure 3:**
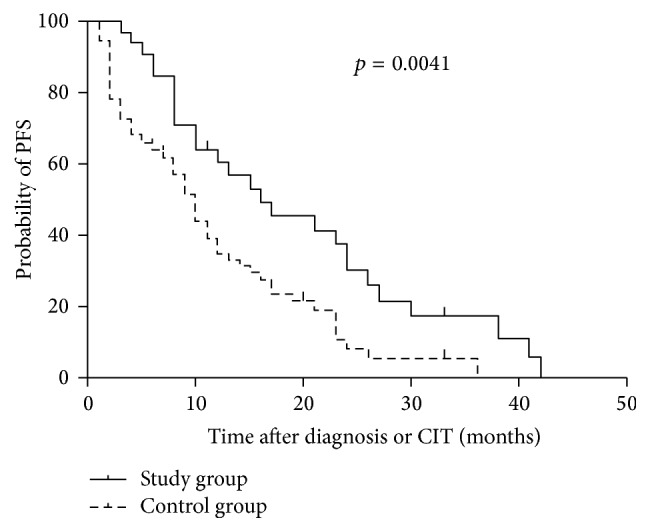
Progression-free survival (PFS) in study and control group. The median PFS in study (*n* = 31) and control group (*n* = 73) was 16 and 10 months, respectively (*p* = 0.0041).

The deadline of follow-up was November 2015 and the median follow-up duration was 10 months (1–42 months), with 18 censored data entries (5 in the study group and 13 in the control group). The study group showed better PFS, with a median PFS of 16 months compared to 10 months for the control group (*p* = 0.004; Figure 3). Using the log-rank statistical test (Mantel-Cox) regression model, with CIT and staging as individual predictors of PFS, only CIT emerged as an independent prognostic factor for PFS (hazard ratio, 0.422; *p* = 0.005). We also evaluated the relationship between viral load change and PFS. The change of viral load for patients receiving CIT therapy was assigned to “increase,” “stable,” and “decrease” subgroups as defined previously. There was no significant correlation between viral load change and PFS using the Spearman test (*p* = 0.453).

### 3.5. Adverse Events

About half of patients underwent mild adverse events (grades 1-2) of ALT and AST in both groups, with mainly distribution of grade 1 (about 40%). Only 2.7% patients had grade 3 adverse events in control group when evaluating AST. It appears to be no obvious difference in the two groups. More detailed data were shown in Table 5. For study and control group, the median ALT level after treatment was 46 IU/mL (range, 14–176 IU/mL) and 56 IU/mL (range, 15–232 IU/mL), respectively, which was not significantly different (*p* = 0.087), respectively. There were no significant differences in the change of AST (median change −3 versus 3, *p* = 0.139) and ALT (median change −3 versus 0, *p* = 0.443) between the two groups (Table 2).

Only three patients (3/31, 9.7%) developed moderate fever after infusion, with temperature ranging from 38.2 to 38.5°C (grade 1), and they all recovered within one hour after receiving oral nonsteroidal anti-inflammatory drugs.

## 4. Discussion

Simultaneous infection of chronic hepatitis C increases the rate of progression and imposes a great challenge for the treatment of patients with HCC. Chronic HCV infection attenuates both the innate and adaptive immune responses, thereby reducing the likelihood of viral clearance as well as the degree of immune-mediated liver injury to allow for coexistence of both the virus and host. HCV thus outpaces the rate of host immune responses and its elimination requires activation of all aspects of immunity. Furthermore, interaction between immune cells is also critical for the control of cancer* in vivo* as well, so that it will have more chance to progress in a patient with an immune system compromised by HCV. Therefore, it is a rational and novel ideal for application of multiple kinds of immunocytes rather than single kind of immunocytes. The results of this retrospective study also showed the significance of this regimen over previous studies on CIT with single type of cells. Previous study showed that CIT with CIK cells alone could prolong PFS in HCC patients [19] but failed to decrease the virus load [20]. Our study showed not only survival benefit of the CIT with three kinds of immunocytes, but also its function for virus control, owing to the synergistic potential of these immune cell types.

In order to control confounding factors to examine the antivirus effect of CIT alone as much as possible, we excluded patients that were coinfected with HBV and HIV because of their potential interaction and defective immunity. Sorafenib users were also excluded because of its evident antitumor and possible anti-HCV effect [21, 22]. Thus, CIT was the sole intervention for viral replication in our study. To evaluate the actual change in viral load for each patient, we adopted the definition of viral load change criteria in other studies. That is, the definition of virus load change in responder patients is defined as experiencing more than a 0.5 log viral load reduction [23]. Minor change and no changes are defined as 0.5–1.0 log reduction and <0.5 log reduction [24]. In our study, we also considered that a change of over 0.5 log was meaningful, since the normal error range is ±0.45 log according to the instruction of the Hepatitis C Virus RNA Diagnostic Kit (Real-Time PCR). The overall viral load decreased in the range of 0.5–2 log, showing good potential of this regimen, since CIT could prevent the increase of viral load during the course of disease in HCC patients with HCV who have no other option for virus control. At the same time, the ideal end-point of HCV treatment is to acquire a sustained virological response. Therefore, there is a relatively big gap of this standard comparing with the ideal end-point. Nevertheless, the moderate virus clearance effects observed suggest that an intrahepatic resistance environment also plays an important role in the performance of CIT.

To further determine the clinical significance of moderate suppression of HCV replication through CIT, such as decreased recurrence of cancer or prolonged survival, survival curves were plotted, and the study group showed better PFS. Previous studies have also shown that CIK cells alone can prolong PFS or overall survival for HCC directly through its antitumor effect [25–28]. We speculate that CIT might have a double impact on virus replication and provide a direct PFS benefit, given that the decrease of virus titer does not appear to contribute to the prolongation of PFS. However, this effect might be due to the insufficient decline in viral load for effective comparison or small sample size; therefore, this association warrants further study.

Our present strategy also showed good efficacy when comparing with other immunotherapies, such as vaccines, and use of immunomodulatory antibodies. A phase I clinical trial using a vaccine in which monocyte-derived dendritic cells were loaded and activated* ex vivo* with lipopeptides also failed to influence viral load [29]. This suggested that some patients may have a reaction to current vaccines and the antivirus effect is still limited. The programmed cell death-1 (PD-1) pathway plays an important role in T cell exhaustion and dysfunction; thus the PD-1 antibody was tested as a candidate immunomodulatory antibody in chimpanzees with HCV. A significant reduction in HCV viremia was observed in one of three treated animals. However, viremia rebounded in the responder animals when the antibody treatment was discontinued [30]. This study suggests that although a clear antivirus effect was observed, only a subsample of subjects might show a reaction, and the effect is transient. According to our results, the combination of CIT showed a moderate and long-lasting antivirus effect and also showed antitumor potential.

We also tried to explore factors that may have an influence on treatment efficacy and found the tendency of female patients achieving better virus control though no significant difference was obtained. In the group showing a decrease of HCV RNA, 5 of 8 patients were female. Although given the small sample size this finding cannot lead to a definitive conclusion, the general trend is certainly worthy of further exploration. It is well established that females usually have a good response to interferon-*γ* (IFN-*γ*) treatment, which may imply that CIT could function via IFN-*γ*. One female patient (72 years) who received 11 courses of CIT showed a decline in HCV RNA levels of over 2 log (from 2.3 × 10^7^ to 1.97 × 10^5^) within 34 months. This may suggest that the number of treatment courses could have a positive effect on the degree of HCV decline. However, the effect of HCV genotype on CIT response remains to be determined, because different genotypes show various responses to interferon. For example, IFN-*γ* is effective in approximately half of patients chronically infected with genotypes 2 and 3 but is much less effective in patients infected with genotypes 1 and 4 [31, 32].

Liver injury secondary to HCV infection is considered to be immune-mediated and not to result from the direct cytopathic effects of the virus [33]. Thus, efforts to enhance host antiviral immunity may theoretically act to promote liver injury. However, in our study, the liver function was found to be stable after CIT, suggesting that it may not be directly involved in liver damage.

Transient fever was the only severe side effect observed in patients, which was readily relieved by nonsteroidal anti-inflammatory drugs. In fact, our results showed that immunotherapy may ameliorate some symptoms: patients reported an increased appetite, improved sleep, gained body weight, and pain relief.

Taken together, our data demonstrate that a combination of innate immune cells could suppress virus replication and preserve the liver function of HCC patients with HCV infection. Simultaneously, the immune cells could perform their antitumor function and prolong the PFS of these patients. Thus, this study provides evidence that the CIT is safe and effective in the treatment of HCC with HCV, highlighting the importance and need to perform a prospective study for this treatment.

## Figures and Tables

**Table 1:** The baseline characteristics of patients.

Characteristics	Control group *n* (%)	Study group *n* (%)	*χ* ^2^	*p* value
Age (y)			0.029	0.866
≤65	39 (53%)	16 (52%)		
>65	34	15		
Gender (M/F)			0.006	0.937
Male	43 (59%)	18 (58%)		
Female	30	13		
BCLC staging			4.964	0.174
0	10	2		
A	42 (58%)	14 (45%)		
B	17	10		
C	4	5		
Conventional therapy			4.935	0.085
RFA	57 (78%)	24 (77%)		
TACE	6	6		
Surgery	10	1		

**Table 2:** The comparison of baseline and the change of ASL/ALT level.

	Control group (IU/mL) median (range)	Study group (IU/mL) median (range)	*p* value
AST1	56 (20–313)	49 (21–102)	0.215
ALT1	56 (15–232)	46 (14–176)	0.087
AST2-AST1	3 (−264–283)	−3 (−43–42)	0.139
ALT2-ALT1	0 (−211–376)	−3 (−94–81)	0.443

Note: AST1/ALT1 as the first point of detection before treatment and AST2/ALT2 as the second point of detection after treatment.

The change of liver function was measured according to deviation in these values (denoted as “AST2-ASL1” or “ALT2-ALT1”). ALT: alanine aminotransferase; AST: aspartate aminotransferase.

**Table 3:** The effects of CIT on levels of virus load in HCC.

	Decrease		Stable		Increase	
	↓>1 log	↓0.5–1 log	↓0–0.5 log	↑0–0.5 log	↑0.5–1 log	↑1 log
Control group (*n* = 68)	5 (7%)	9 (13%)	18 (26%)	19 (28%)	8 (12%)	9 (13%)
Study group (*n* = 28)	4 (14%)	4 (14%)	9 (32%)	11 (39%)	0 (0%)	0 (0%)

**Table 4:** The effects of CIT on distribution of virus load in HCC.

	Decrease	Stable	Increase	*χ* ^2^	*p* value
Control group (*n* = 68)	14 (21%)	37 (54%)	17 (25%)	8.519	0.014
Study group (*n* = 28)	8 (29%)	20 (71%)	0 (0%)		

**Table 5:** The distribution of adverse events of ASL2/ALT2 in the two groups.

	Grade 1	Grade 2	Grade 3
AST2 in control (*n* = 73)	29 (39.7%)	14 (19.2%)	2 (2.7%)
AST2 in study (*n* = 31)	15 (48.4%)	3 (9.6%)	0 (0.0%)
ALT2 in control (*n* = 73)	30 (41.1%)	9 (12.3%)	0 (0.0%)
ALT2 in study (*n* = 31)	8 (25.8%)	2 (6.5%)	0 (0.0%)
