# Corrigendum to “Dual Effects of Cellular Immunotherapy in Inhibition of Virus Replication and Prolongation of Survival in HCV-Positive Hepatocellular Carcinoma Patients”

**DOI:** 10.1155/2022/9761463

**Published:** 2022-07-16

**Authors:** Lei Qian, Nanya Wang, Huimin Tian, Haofan Jin, Hengjun Zhao, Chao Niu, Hua He, Tingwen Ge, Wei Han, Jifan Hu, Dan Li, Fujun Han, Jianting Xu, Xiao Ding, Jingtao Chen, Wei Li, Jiuwei Cui

**Affiliations:** ^1^Department of Cancer Center, The First Hospital of Jilin University, Changchun 130021, China; ^2^Institute of Translational Medicine, The First Hospital of Jilin University, Changchun 130021, China

In the article titled “Dual Effects of Cellular Immunotherapy in Inhibition of Virus Replication and Prolongation of Survival in HCV-Positive Hepatocellular Carcinoma Patients” [1], the authors wish to correct Figure 2. Due to an oversight when preparing the manuscript, it is duplicated with Figure 3 in a previous publication by the authors [2]. These two studies were related, and the data were saved to the same location, contributing to the erroneous selection of the image for publication. The authors apologise for this error and provide the corrected Figure 2 as follows:

## Figures and Tables

**Figure 1:**
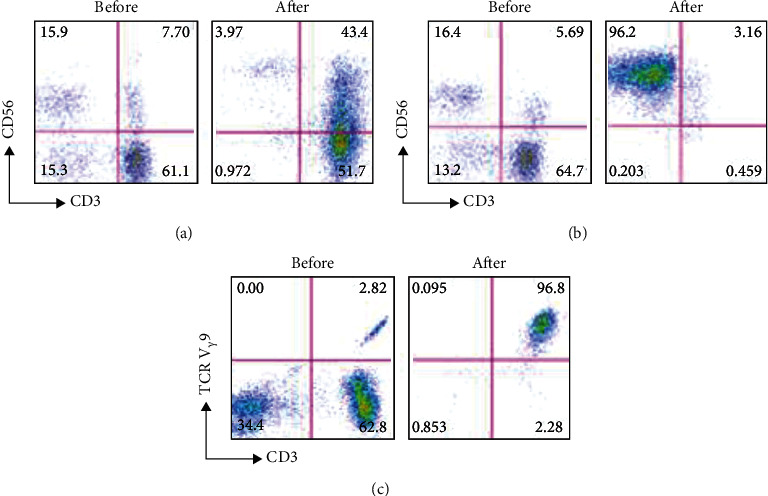
(a) The percentage of CIK cells including (CD3^+^ CD56^+^), (CD3^+^ CD56^−^), and (CD3^−^ CD56^+^) before and after induction and CD4^+^ and CD8^+^ before and after induction in one of the patients. (b) The percentage of NK cells (CD3^+^ CD56^−^) before and after induction and the activated NK (CD56^+^ CD69^+^) before and after induction in one of the patients. (c) The percentage of *γδ*T before and after induction in one of the patients.
